# A comparison of extended right hemicolectomy and transverse colectomy with D3 lymph node dissection for pathological stage II/III advanced mid-transverse colon cancer

**DOI:** 10.1007/s00384-026-05202-x

**Published:** 2026-07-29

**Authors:** Emi Ota, Mayumi Ozawa, Kazuya Nakagawa, Hirokazu Suwa, Yusuke Suwa, Itaru Endo

**Affiliations:** 1 Department of Surgery, Yokosuka Kyosai Hospital, Yokosuka, Japan; 2 Department of Surgery, Gastroenterological Center, Yokohama City University Medical Center, Yokohama, Japan; 3 Department of Gastroenterological Surgery, Yokohama City University Graduate School of Medicine, Yokohama, Japan

**Keywords:** Mid-transverse colon cancer, Extended right hemicolectomy, Transverse colectomy, D3 lymph node dissection, Middle colic artery

## Abstract

**Purpose:**

To date, no study has focused exclusively on D3 lymph node dissection for mid-transverse colon cancer. The present study compared surgical and oncological results following extended right hemicolectomy (exRHC) and transverse colectomy (TC) with standardized D3 lymphadenectomy in patients with locally advanced mid-transverse colon cancer.

**Methods:**

Patients with stage II or III mid-transverse colon cancer who underwent D3 lymphadenectomy with central ligation of the middle colic artery (MCA) between January 2013 and December 2022 were divided into two groups by surgical procedure. We compared patient short- and long-term outcomes between the groups.

**Results:**

Eighty-three patients (exRHC, *n* = 56; TC, *n* = 27) were analyzed. Overall complication rates (35.7% vs. 22.2%, *p* = 0.458) and Clavien-Dindo grade ≥ 3 complications (10.7% vs. 3.7%, *p* = 0.418) did not differ significantly between the exRHC and TC groups. In contrast, postoperative ileus occurred more frequently in the exRHC group (16.1% vs. 0%, *p* = 0.028), and the hospitalization duration was significantly longer in the exRHC group (8 vs. 7 days, *p* = 0.016). The 3-year relapse free survival (RFS) was 86.3% for exRHC and 80.3% for TC (*p* = 0.63), and the 3-year overall survival (OS) was 92.6% and 91.6%, respectively (*p* = 0.778).

**Conclusions:**

In advanced mid-transverse colon cancer with D3 lymph node dissection and central MCA ligation, exRHC associated with a higher risk of postoperative ileus and longer hospital stay than TC. Given similar oncologic outcomes, TC may be a more favorable surgical option owing to its faster recovery.

**Supplementary Information:**

The online version contains supplementary material available at https://doi.org/10.1007/s00384-026-05202-x.

## Introduction

Transverse colon cancer is defined as cancer occurring in the hepatic flexure, mid-transverse colon, or splenic flexure [1], and it accounts for 10% of all colorectal cancers [2, 3]. Currently, there is no consensus regarding standard surgical procedures for mid-transverse colon cancer. In clinical practice, the choice of surgical strategy between extended right hemicolectomy (exRHC) and transverse colectomy (TC) is often based on surgeon preference. Despite variability in technique, exRHC remains the most commonly performed procedure for mid-transverse colon cancer [4–6].

Several studies have demonstrated that TC for mid-transverse colon cancer is associated with a higher incidence of postoperative complications [6–8] and higher recurrence rates than exRHC [5, 9]. However, these findings are confounded by staging imbalances and are inconsistent with the extent of lymphadenectomy protocols. In fact, some retrospective studies have shown that patients who underwent TC had significantly earlier UICC stages and fewer high-risk pathological features than those who underwent exRHC [4, 8, 10], making a direct comparison difficult. Some studies have compared extended hemicolectomy and TC [4, 8]; in clinical practice for mid-transverse colon cancer, the surgical choice is typically exRHC or TC. Extended left hemicolectomy is less commonly selected unless the tumor is located toward the left side of the transverse colon.

Based on the Japanese Society for Cancer of the Colon and Rectum (JSCCR) [11], D3 lymphadenectomy is defined as central ligation of the feeding vessels at their origin with complete excision of the mesocolon, including removal of main lymph nodes along the primary feeding arteries. D3 lymph node dissection has been reported to reduce the risk of local recurrence and improve survival. However, because of the increased technical complexity, exposure of critical retroperitoneal organs—such as the duodenum, pancreas, and mesenteric root—may increase the risk of serious intraoperative complications [12]. Despite the fact that surgical difficulty varies according to the extent of lymphadenectomy even within the same operative procedure, prior comparative studies of exRHC and TC have included heterogeneous lymphadenectomy protocols, often mixing D1, D2, and D3 dissections, thereby confounding the evaluation of true surgical and oncological outcomes.

To our knowledge, no studies have focused exclusively on D3 lymphadenectomy for mid-transverse colon cancer. In the present study, we compared the surgical and oncological outcomes of exRHC and TC restricted to patients with locally advanced mid-transverse colon cancer who uniformly underwent D3 lymph node dissection with central ligation of the middle colic artery (MCA).

## Methods

### Patient selection

A retrospective observational analysis involving multiple centers was undertaken at Yokosuka Kyosai Hospital, Yokohama City University Medical Center, and Yokohama City University Hospital. Ethical approval for this research was obtained from the institutional review board (approval ID. B180400017). The investigation was carried out in compliance with internationally recognized ethical standards, including the Declaration of Helsinki. Prior to participation, all patients provided written consent. This manuscript was prepared in compliance with the Strengthening the Reporting of Observational Studies in Epidemiology (STROBE) recommendations for reporting observational research.

Patients requiring mid-transverse colon cancer surgery from January 2013 to December 2022 were included in this study. To minimize selection bias, patients were enrolled consecutively from the database. The study enrolled patients who satisfied the following inclusion criteria: (1) patients with primary mid-transverse colon cancer; (2) those who were diagnosed with pathological stage II or III; and (3) those who underwent surgery for exRHC or TC with D3 lymph node dissection with ligation of the MCA. Patients with diseases other than primary tumors (including tumor recurrence, neuroendocrine neoplasms and diverticula), pathological stage I or IV, concomitant cancers other than transverse colon cancer, combined organ resection, right or left transverse colon, right hemicolectomy, left hemicolectomy, D1 or D2 lymph node dissection, without anastomosis, or incomplete data were excluded from this study.

### Definition of mid-transverse colon cancer

For anatomical classification, the transverse colon was trisected between the hepatic and splenic flexures; lesions identified in the central segment were designated as mid-transverse colon cancer. Tumor location was clinically assessed by the operating surgeon using preoperative imaging modalities—such as colonography, barium enema examination, and computed tomography—as well as intraoperative observations. The final tumor location was determined based on intraoperative findings.

### Definition of exRHC and TC

ExRHC involved ligation of the ileocolic artery (ICA) and root of the MCA [13]. TC was defined as a procedure involving ligation of the MCA at the root while preserving both the ICA and right colic artery (RCA). The resection included approximately 10 cm of normal bowel on either side of the tumor. Complete D3 lymphadenectomy was performed, which referred to the removal of lymph nodes up to the root of the MCA (classified as 223 by the JSCCR) [11]. The choice of surgical approach (open or laparoscopic), procedure (exRHC or TC) and anastomosis methods (end-to-end, side-to-side, and functional end-to-end anastomosis) were at the discretion of the expert colorectal specialists at each institution based on individual cases. Expert colorectal specialists certified by the Japan Society of Laparoscopic Colorectal Surgery (JSLCS) always participate in the surgery at all three institutions, thereby ensuring a consistent quality of surgery.

### Definition of lymph node dissection

Lymphadenectomy was performed according to the JSCCR guidelines [11]. The lymph nodes were divided into three categories: main lymph nodes (MLNs), intermediate lymph nodes (ILNs), and pericolic lymph nodes (PLNs). MLNs were defined as those located at the root of the feeding artery, ILNs were those located along the feeding artery, and PLNs were those situated along the marginal artery and adjacent to the bowel wall. D3 lymphadenectomy included dissection of the MLNs, ILNs, and PLNs; D2 lymphadenectomy included dissection of the LLNs and ILNs; and D1 lymphadenectomy was limited to dissection of the PLNs. D3 lymphadenectomy is defined as dissection with ligation of the feeding vessels at their roots, accompanied by complete mesocolic excision. D3 lymphadenectomy was performed based on preoperative imaging findings and intraoperative identification or the vascular root.

### Endpoints

The primary endpoint was postoperative ileus. The secondary endpoints included overall postoperative complications, major complications (Clavien-Dindo grade 3), postoperative length of hospital stay, number of harvested lymph nodes, 3-year relapse-free survival (RFS), and 3-year overall survival (OS).

### Observed indicators

Clinical and demographic data were collected to provide the characteristics of the study population. These included age, sex, body mass index (BMI), American Society of Anesthesiologists Physical Status Classification System (ASA-PS), comorbidities, histological type, tumor diameter, number of harvested lymph nodes, proximal and distal resection margins, lymphovascular invasion, p-T stage, p-N stage, and p-Stage. A histological examination of the tumor was performed according to the World Health Organization (WHO) classification system [14]. Clinical and pathological staging were determined using the 8th edition of the TNM classification by the Union for International Cancer Control (UICC) [15]. Operative data included operative time, blood loss, approach, type of anastomosis, and maximum wound length. Robot surgery for colon cancer became covered by the national health insurance system in Japan from April 2022. Postoperative data included the length of postoperative stay, complications, reoperation, adjuvant chemotherapy, recurrence rate, 3-year RFS, and 3-year OS. The severity of complications was evaluated using the CD classification [16]. Postoperative ileus was identified by retrospective review of medical records. It was diagnosed based on documented clinical symptoms and radiologic findings, and was defined as cases requiring therapeutic interventions, including fasting, nasogastric tube placement, or long intestinal tube insertion. All three institutions used a clinical pathway, oral intake was initiated on postoperative day 2, and patients were discharged on postoperative day 7 if blood tests and radiographic findings on postoperative day 5 showed no abnormalities. ERAS protocols were not implemented at any of the participating institutions during the study period. For patients with p-Stage III, we administered adjuvant chemotherapy to those aged <80 years with an ECOG performance status of 0–1 who were considered fit to tolerate chemotherapy. The date of censoring was defined as the last date on which follow-up information was confirmed in the medical records. Tumor recurrence was defined as the date on which recurrence was confirmed on radiological images. Because the number of patients with mid-transverse colon cancer was limited, we used 3-year RFS and OS for evaluation to allow analysis in as many cases as possible. Three-year DFS is an established surrogate marker for 5-year OS in colorectal cancer [17].

### Statistical analyses

Continuous data were summarized using median values. All statistical analyses were carried out with the EZR software (version 3.0; Saitama Medical Center, Jichi Medical University, Saitama, Japan). Categorical variables were conducted with either Fisher’s exact test or the chi-squared test, while continuous variables were assessed using the Mann–Whitney *U* test. The 3-year RFS and OS rates since the primary surgery were calculated using the Kaplan–Meier method. Multivariate analysis and propensity score matching (PSM) was conducted to balance the c-Stage between the groups, and the robustness of the primary findings was assessed in a sensitivity analysis.

## Results

### Patients’ background characteristics

**Fig. 1:**
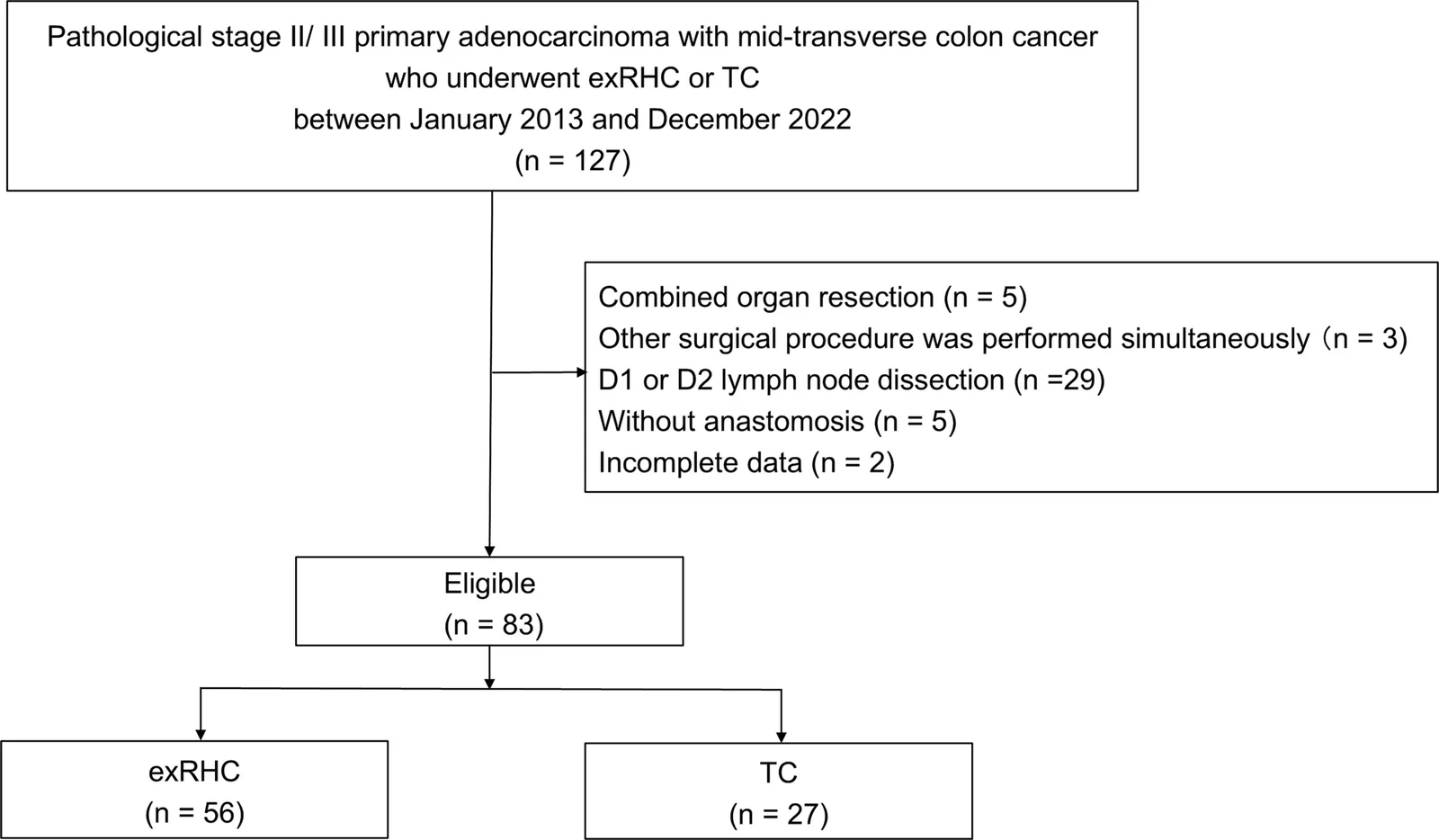
Flowchart of patient selection

Figure 1 shows the patient flow chart. A total of 83 patients from 3 institutions were included and analyzed in this study. Among them, 56 underwent exRHC, and 27 underwent TC. Table 1 shows the patient backgrounds for exRHC and TC. The median patient age was 72 years old. Of the 83 patients, 44 (53.0%) were male, and 39 (47.0%) were female. There were no significant differences in the BMI, ASA-PS, or comorbidities between the groups. There were also no marked differences in the rates of c-T stage and c-N stage between the groups. Regarding c-Stage, the proportion of c-Stage III was higher in the exRHC group than in the TC group (69.6% and 44.4%, respectively).

**Table 1:** Background of patients receiving exRHC and TC

	All (*n* = 83)	exRHC (*n* = 56)	TC (*n* = 27)	*p*
Age, median (IQR), years	72 (67–77)	73 (67.8–77)	70 (67–77)	0.543
Sex				
Male	44 (53.0%)	35 (62.5%)	9 (33.3%)	0.019
Female	39 (47.0%)	21 (37.5%)	18 (66.7%)	
BMI, median (IQR), kg/m^2^	21.5 (18.9–23.9)	21.7 (19.1–23.5)	20.0 (18.7–24.5)	0.737
ASA-PS score				1
I	11 (13.3%)	7 (12.5%)	4 (14.8%)	
II	65 (78.3%)	44 (78.6%)	21 (77.8%)	
III	7 (8.4%)	5 (8.9%)	2 (7.4%)	
IV	0 (0%)	0 (0%)	0 (0%)	
Hypertension				0.358
No	46 (55.4%)	29 (51.8%)	17 (63.0%)	
Yes	37 (44.6%)	27 (48.2%)	10 (37.0%)	
Cardiac disease				0.49
No	72 (86.7%)	47 (83.9%)	25 (92.6%)	
Yes	11 (13.3%)	9 (16.1%)	2 (7.4%)	
Diabetes				1
No	69 (83.1%)	46 (82.1%)	23 (85.2%)	
Yes	14 (16.9%)	10 (17.9%)	4 (14.8%)	
c-T stage				0.353
T1	3 (3.6%)	1 (1.8%)	2 (7.4%)	
T2	9 (10.8%)	6 (10.7%)	3 (11.1%)	
T3	41 (49.4%)	26 (46.4%)	15 (55.6%)	
T4	30 (36.1%)	23 (41.1%)	7 (25.9%)	
c-N stage				0.107
N0	32 (38.6%)	17 (30.4%)	15 (55.6%)	
N1	38 (45.8%)	29 (51.8%)	9 (33.3%)	
N2	13 (15.7%)	10 (17.9%)	3 (11.1%)	
c-Stage				0.098
I	9 (10.8%)	5 (8.9%)	4 (14.8%)	
II	23 (27.7%)	12 (21.4%)	11 (40.7%)	
III	51 (61.4%)	39 (69.6%)	12 (44.4%)	

*exRHC* extended right hemicolectomy, *TC* transverse colectomy, *IQR* interquartile range, *BMI* body mass index, *ASA-PS* American Society of Anesthesiologists Physical Status Classification System

### Operative details and pathological outcomes

Table 2 shows the operative details and pathological outcomes. There were no marked differences in the exRHC and TC groups in operative time (227.5 min and 213.0 min, respectively; *p* = 0.296), blood loss (14.5 ml and 9.0 ml, respectively; *p* = 0.592), surgical approaches, type of anastomosis, site of anastomosis, or maximum wound length. The postoperative length of stay was significantly longer in the exRHC than in the TC group (8 and 7 days, respectively; *p* = 0.016). There were no marked differences between the exRHC and TC groups in histological type, tumor diameter (50 and 50 mm, respectively; *p* = 0.108), lymphatic invasions, and venous invasions. p-T stage, p-N stage, or p-Stage (p-Stage II:III, 51.8%:48.1% and 48:2%:51.9%, respectively; *p* = 0.817). The median number of harvested lymph nodes was significantly higher in the exRHC than in the TC group (38.5 and 26.0, respectively; *p* < 0001). However, there was no significant difference in the number of metastatic lymph nodes between the exRHC and TC groups (0 and 1, respectively; *p* = 1). There was also no significant difference in the number of harvested lymph nodes at the root of the MCA (223) between the exRHC and TC groups (4 and 4, respectively; *p* = 1). The proximal margin was significantly longer (250 and 95 mm, respectively; *p* < 0.001), whereas the distal margin was significantly shorter (79 and 90 mm, respectively; *p* = 0.031) in the exRHC than in the TC group. Table 3 shows the number of harvested and metastatic lymph nodes in the ICA and RCA territories. No lymph node metastasis was observed in the ICA region, whereas one metastatic lymph node was identified in each of stations #211 and #212 in the RCA region. The pathological characteristics of the two cases were as follows: one case had tub2, pT3N2, lymphatic invasion positive, venous invasion negative, and p-Stage IIIC, while the other had tub2, pT3N1, lymphatic invasion negative, venous invasion positive, and p-Stage IIIB. A review of the operative records revealed that there were no cases in the TC group in which the RCA branched from the MCA.

**Table 2:** Operative details and pathological outcomes of exRHC and TC

	All (*n* = 83)	exRHC (*n* = 56)	TC (*n* = 27)	*p*
Operative time, median (IQR), min	224 (189.5–271)	227.5 (196.5–274.3)	213.0 (178.0–257.5)	0.296
Blood loss, median (IQR), mL	13 (0–69)	14.5 (0–76.3)	9.0 (0–36.0)	0.592
Surgical approaches				0.869
Open surgery	9 (10.8%)	6 (10.7%)	3 (11.1%)	
Conventional laparoscopic surgery	72 (86.7%)	49 (87.5%)	23 (85.2%)	
Robotic-assisted laparoscopic surgery	2 (2.4%)	1 (1.8%)	1 (3.7%)	
Types of anastomoses				0.097
End-to-end anastomosis	22 (26.5%)	11 (19.6%)	11 (40.7%)	
Side-to-end anastomosis	5 (6.0%)	3 (5.4%)	2 (7.4%)	
Functional end-to-end anastomosis	56 (67.5%)	42 (75%)	14 (51.9%)	
Site of anastomosis				0.49
Intracorporeal anastomosis	11 (13.3%)	9 (16.1%)	2 (7.4%)	
Extracorporeal anastomosis	72 (86.7%)	47 (83.9%)	25 (92.6%)	
Maximum wound length, median (IQR), mm	50 (40–60)	50 (44.3–62.5)	45 (40–60)	0.34
Postoperative length of stay, median (IQR), days	8 (6–11.5)	8 (7–14.3)	7 (6–10)	0.016
Histological type				0.216
Well/moderately differentiated adenocarcinoma	75 (90.4%)	51 (91.1%)	24 (88.9%)	
Poorly differentiated adenocarcinoma	3 (3.6%)	3 (5.4%)	0 (0%)	
Mucinous adenocarcinoma	5 (6.0%)	2 (3.6%)	3 (11.1%)	
Tumor diameter, median (IQR), mm	50 (35.5–62.5)	50 (40–70)	50 (31–55)	0.108
Number of harvested lymph nodes, median (IQR)	34 (26.5–46)	38.5 (29.0–50.3)	26.0 (15.5–33.0)	<0.001
Number of harvested lymph nodes at the root of MCA (223), median (IQR)	4 (3–7)	4 (3–6)	4 (1–8.5)	1
Number of metastatic lymph nodes, median (IQR)	0 (1–2)	0 (0–2)	1 (0–1.5)	1
Number of metastatic lymph nodes at the root of MCA (223), median (IQR)	0 (0–0)	0 (0–0)	0 (0–0)	0.346
Number of metastatic lymph nodes at the root of MCA (223), *n* (%)	6 (7.2%)	3 (5.4%)	3 (11.1%)	0.358
Proximal margin (PM), median (IQR), mm	210 (112.5–274)	250 (204.8–310.3)	95 (80–121.5)	<0.001
Distal margin (DM), median (IQR), mm	80 (65–95)	79 (58.8–88.5)	90 (74.0–100)	0.031
Lymphatic invasion				0.81
Negative	30 (36.1%)	21 (37.5%)	9 (33.3%)	
Positive	53 (63.9%)	35 (63.5%)	18 (66.7%)	
Venous invasion				0.605
Negative	22 (26.5%)	16 (28.6%)	6 (22.2%)	
Positive	61 (73.5%)	40 (71.4%)	21 (77.8%)	
p–T stage				0.551
T1	1 (1.2%)	0 (0%)	1 (3.7%)	
T2	1 (1.2%)	1 (1.8%)	0 (0%)	
T3	56 (67.5%)	37 (66.1%)	19 (70.4%)	
T4	25 (30.1%)	18 (32.1%)	7 (25.9%)	
p-N stage				0.944
N0	42 (50.6%)	29 (51.8%)	13 (48.1%)	
N1	32 (38.6%)	21 (37.5%)	11 (40.7%)	
N2	9 (10.8%)	6 (10.7%)	3 (11.1%)	
p-Stage				0.817
II	42 (50.6%)	29 (51.8%)	13 (48.1%)	
III	41 (49.4%)	27 (48.2%)	14 (51.9%)	

*exRHC* extended right hemicolectomy, *TC* transverse colectomy, *IQR* interquartile range, *MCA* middle colic artery

**Table 3:** Number of harvested lymph nodes and metastatic lymph nodes of exRHC (*n* = 56)

	Number of harvested lymph nodes, median (IQR)	Number of metastatic lymph nodes, *n* (%)
Number of harvested lymph nodes at #201	7 (4–9.5)	0 (0%)
Number of harvested lymph nodes at #202	4 (2–6)	0 (0%)
Number of harvested lymph nodes at #203	3 (2–5)	0 (0%)
Number of harvested lymph nodes at #211	3 (2–6)	1 (1.8%)
Number of harvested lymph nodes at #212	2.5 (1–4)	1 (1.8%)
Number of harvested lymph nodes at #213	2.5 (1–4.3)	0 (0%)

*exRHC* extended right hemicolectomy, *IQR* interquartile range

### Postoperative short-term outcomes

Table 4 presents the short-term outcomes of the study. There were no significant differences in the overall complication rate (35.7% and 22.2%, respectively; *p* = 0.458) or in the incidence of CD grade ≥ 3 complications (10.7% and 3.7%, respectively; *p* = 0.418) between the exRHC and TC groups. However, the incidence of postoperative ileus was significantly higher in the exRHC group than in the TC group (16.1% and 0%, respectively; *p* = 0.028). Among the 9 patients who developed postoperative ileus, 5 required nasogastric tube placement, 2 required long-tube decompression, and 2 were managed with fasting alone. There was no statistically significant difference in the rates of adjuvant chemotherapy between the exRHC and TC groups (30.4% and 44.4%, respectively; *p* = 0.228). Reasons for not receiving adjuvant chemotherapy in p-Stage III were as follows: 7 patients were aged 80 years, 2 declined chemotherapy, 1 had a Clavien-Dindo grade ≥ 3 complication, 7 had performance status ≥ 2 (comorbidities such as dementia, schizophrenia, Parkinson’s disease, depression, congestive heart failure, and poorly controlled diabetes mellitus), and the reasons were unknown in 4 patients.

**Table 4:** Postoperative short-term outcomes and long-term outcomes of exRHC and TC

	All (*n* = 83)	exRHC (*n* = 56)	TC (*n* = 27)	*p*
Complications, *n* (%)				
All grade	27 (31.3%)	20 (35.7%)	7 (22.2%)	0.458
Clavien-Dindo grade ≥ 2	17 (20.5%)	13 (23.2%)	4 (14.8%)	0.562
Clavien-Dindo grade ≥ 3	7 (8.4%)	6 (10.7%)	1 (3.7%)	0.418
Complications factors, *n* (%)				
Surgical site infection	8 (9.6%)	5 (8.9%)	3 (11.1%)	0.711
Intraabdominal abscess	4 (4.8%)	3 (5.4%)	1 (3.7%)	1
Ileus	9 (10.8%)	9 (16.1%)	0 (0%)	0.028
Anastomotic leakage	2 (2.4%)	1 (1.8%)	1 (3.7%)	0.547
Diarrhea	7 (8.4%)	6 (10.7%)	1 (3.7%)	0.418
Reoperation, *n* (%)	0 (0%)	0 (0%)	0 (0%)	NA
Adjuvant chemotherapy	29 (34.9%)	17 (30.4%)	12 (44.4%)	0.228

*exRHC* extended right hemicolectomy, *TC* transverse colectomy

Multivariate analysis showed no statistically significant association between the surgical procedure and postoperative ileus (Supplementary Table 1). In contrast, after PSM to balance the clinical stage between the two groups, univariate analysis demonstrated a significantly higher incidence of postoperative ileus in the exRHC group (Supplementary Table 2).

Table 5 shows the recurrence sites. In the TC group, no lymph node recurrence was observed in the ICA or RCA regions.

**Table 5:** Postoperative recurrence site of exRHC and TC

	All (*n* = 83)	exRHC (*n* = 56)	TC (*n* = 27)	*p*
Recurrence site				0.764
Liver	8 (9.6%)	6 (10.7%)	2 (7.4%)	
Pulmonary	3 (3.6%)	1 (1.8%)	2 (7.4%)	
Local recurrence	3 (3.6%)	1 (1.8%)	2 (7.4%)	
Peritoneal dissemination	3 (3.6%)	1 (1.8%)	2 (7.4%)	
Ovary	1 (1.2%)	1 (1.8%)	0 (0%)	
Median follow up period, median (IQR), months	60.5 (38.9–78.7)	58.7 (41.5–80.3)	60.8 (32.3–68.3)	0.662

*exRHC* extended right hemicolectomy, *TC* transverse colectomy

### Long-term outcomes

**Fig. 2:**
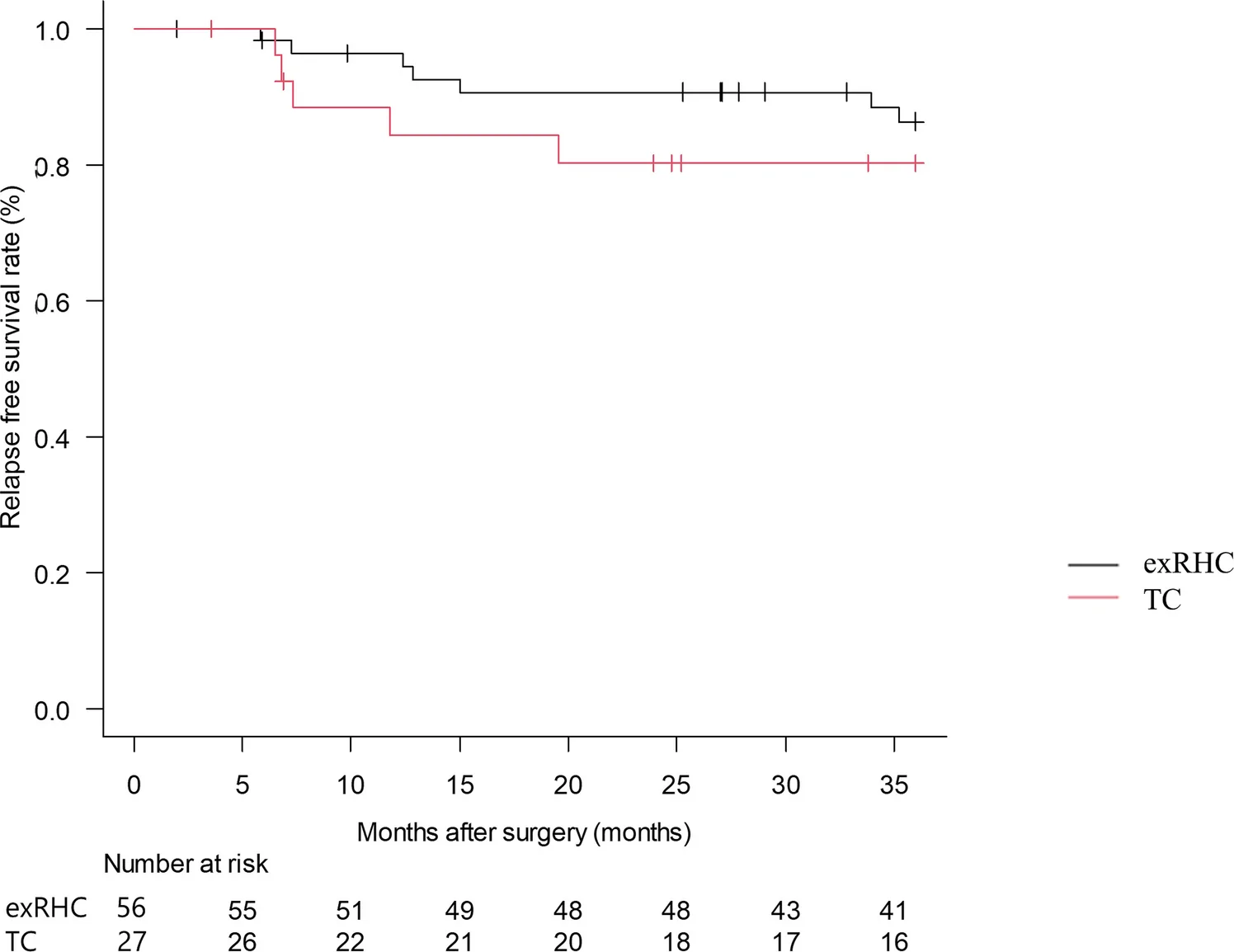
The 3-year relapse free survival (RFS) of mid-transverse colon cancer patients treated by exRHC or TC. RFS was defined as the interval from primary tumor resection to the date of radiologically confirmed recurrence. The *p*-values were calculated using the log-rank test. There were no marked differences in the exRHC and TC groups in the 3-year RFS (86.3% and 80.3%, respectively; *p* = 0.63)

**Fig. 3:**
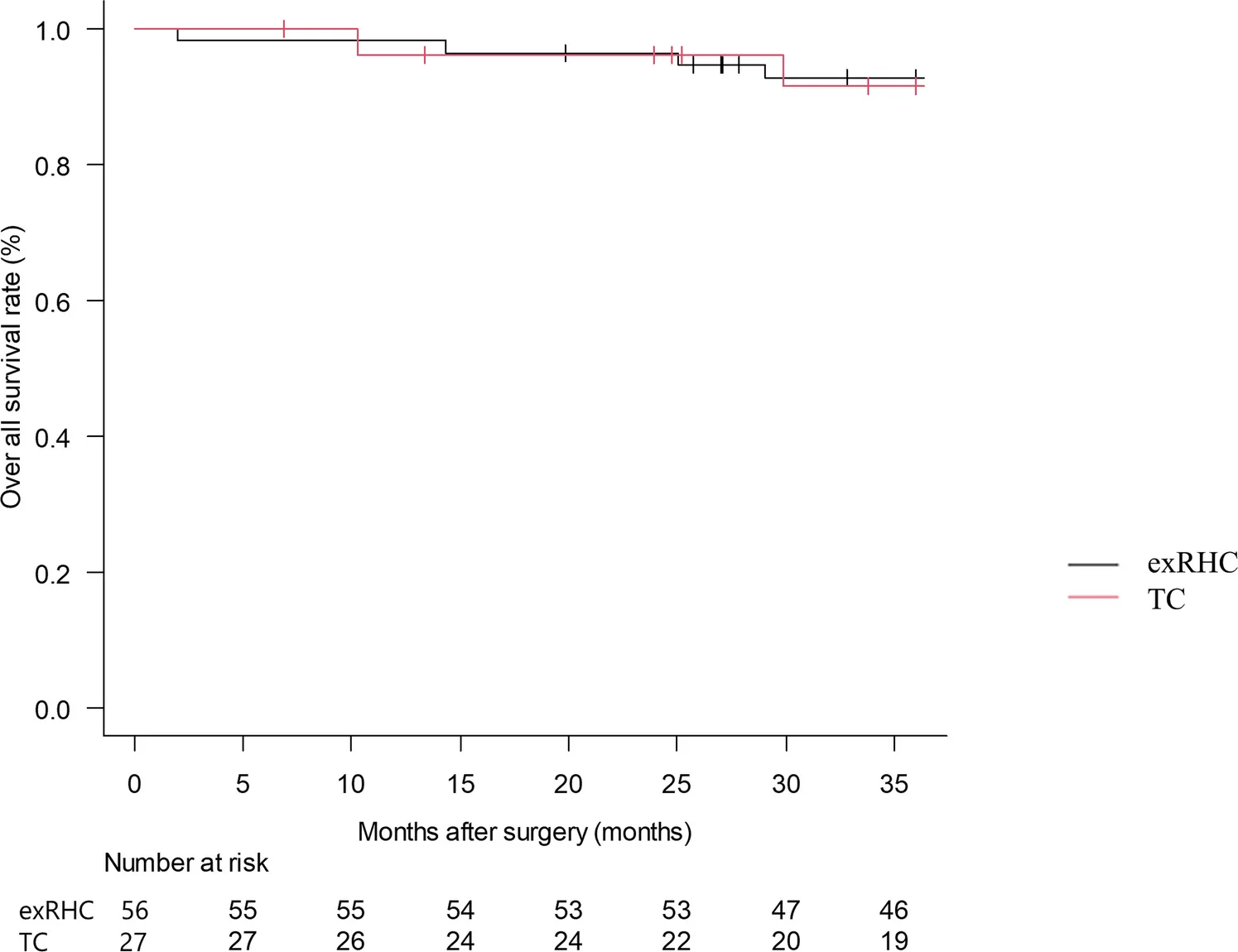
The 3-year overall survival (OS) of mid-transverse colon cancer patients treated by exRHC or TC. OS was defined as the interval from primary tumor resection to the date of death. The *p*-values were calculated using the log-rank test. There were no marked differences in the exRHC and TC groups in the 3-year OS(92.6% and 91.6%, respectively; *p* = 0.778)

There were also no marked differences between the exRHC and TC groups in the 3-year RFS (86.3% and 80.3%, respectively; *p* = 0.63) and 3-year OS (92.6% and 91.6%, respectively; *p* = 0.778), as shown in Figs. 2 and 3. The median follow-up period was 58.7 (41.5–80.3) months vs. 60.8 (32.3–68.3) months, *p* = 0.662, respectively.

There were no statistically significant differences between the hospitals in terms of patients’ background (such as age, sex, ASA-PS score, c-Stage), surgical procedure selection (Yokosuka Kyosai Hospital vs. Yokohama City University Medical Center vs. Yokohama City University Hospital, exRHC; TC, 67.7%; 32.3% vs. 64.7%; 35.3% vs. 72.2%; 27.8%, *p* = 0.954), postoperative ileus (12.9% vs. 11.8% vs. 5.6%, *p* = 0.815), 3-year RFS (83.3% vs. 83.6% vs. 87.2%, *p* = 0.766) and 3-year OS (96.8% vs. 90.6% vs. 87.8%, *p* = 0.499).

## Discussion

The difficulty of surgical techniques for exRHC and TC, as well as postoperative complication rates, are expected to differ significantly depending on the extent of lymphadenectomy and ligation of central blood vessels. However, previous studies have not unified these factors in their investigation. In the present study, we limited our analysis to D3 lymph node dissection in which the MCA was transected at the root. We found that exRHC was associated with a higher incidence of postoperative ileus and prolonged hospitalization than TC. To our knowledge, this is the first report to examine an appropriate procedure for mid-transverse colon cancer with a standardized level of lymphadenectomy.

Postoperative ileus occurred more frequently with a longer hospitalization duration in the exRHC group than in the TC group in our study. One possible reason for the increased incidence of ileus in the exRHC group is the extent of bowel resection. In the exRHC group, the resected proximal bowel was 56 mm longer on average than in the TC group. Nevertheless, the extent of resection itself was not associated with favorable outcomes [18, 19]. Some studies have also recommended a longitudinal resection margin of 10 cm from the tumor, as lymph node spread beyond this distance is extremely rare [20–22]. ExRHC also involves a wider range of bowel mobilization and greater surgical invasion along with extensive bowel resection than TC [21–23]. Second, exRHC requires ligation of the three vessels, whereas TC involves minimal vascular handling, thereby better preserving bowel perfusion. In laparoscopic surgery, exRHC requires cranial or pelvic repositioning of the small intestine and frequent changes in patient positioning. In contrast, TC is often performed with minimal manipulation of the small intestine. Some previous studies have found no significant difference in complication rates between exRHC and TC [8, 23], whereas others, despite lacking statistical significance, reported higher complication rates in exRHC (49.1% vs. 39.5%; 11.4% vs. 3.2%, *p* = 0.086) [7, 24]. These authors suggested that the increased rate of complications may be attributed to the higher incidence of postoperative ileus associated with exRHC than with TC. However, some studies have reported higher complication rates with TC, possibly due to the technical difficulty of mobilizing both the hepatic and splenic flexures, as well as variability in surgeon preference and skill [5, 9, 25]. Third, resection of the ileocecal valve may be involved. The absence of this anatomical barrier may disrupt the functional separation between the small and large intestines, potentially contributing to dysbiosis, small intestinal bacterial overgrowth (SIBO)-like conditions, and impaired gut motility [26, 27]. Fourth, TC has the advantage of preserving the terminal ileum, ascending colon, and ileocecal valve, which may reduce the risk of injury to the autonomic nerve plexus. In contrast, exRHC often involves injury to or resection of the autonomic nerve plexus, which may be a major factor contributing to postoperative diarrhea and impaired bowel function [28–30]. In our study, although the difference was not statistically significant, postoperative diarrhea was more frequent in the exRHC group (10.7% vs. 3.7%, *p* = 0.418).

Many previous studies have reported that exRHC is significantly associated with prolonged operative times than TC [5, 9, 24, 31]. However, some studies have found no significant difference in the operative time between the two procedures [7, 25]. Regarding intraoperative blood loss, most reports indicated no significant differences between the two procedures [5, 23, 25]. However, these prior studies often included cases with varying extents of lymph node dissection (D1–D3), which limits the comparability of their findings. In the present study, which exclusively analyzed cases undergoing D3 lymphadenectomy with MCA root ligation, no significant differences were observed in operative time or blood loss between exRHC and TC.

Although the number of harvested lymph nodes was significantly higher in the exRHC than in the TC group, there were no differences in the number of metastatic lymph nodes or the recurrence rates. In addition, there was no significant difference in the number of harvested lymph nodes at the root of the MCA (223) between the groups. ExRHC is a standard surgical procedure for right-sided colonic disease and typically involves ligation of three vessels: the MCA, RCA, and ICA. Consequently, exRHC is associated with a greater number of harvested lymph nodes, ranging from 24 to 33, than TC, which typically yields 12 to 20 nodes [5, 7, 24, 25, 32]. However, Park et al. reported that the rate of lymph node metastasis to the RCA was 10% in mid-transverse colon cancer, whereas no metastasis was observed in the ICA [8]. Similarly, Matsuda et al. found no metastases in either the RCA or ICA lymph nodes [25]. Milone et al. and Almoregy et al. also reported that, although more lymph nodes were harvested in the exRHC, the number of positive lymph nodes was comparable to that in the TC group [5, 9]. Fukuoka et al. suggested that while RHC including RCA dissection may be appropriate for right-sided transverse colon cancer, extensive lymphadenectomy, particularly including both the ICA and RCA, may be excessive for central transverse colon cancer. They argued that this approach may simply reflect adherence to the conventional principles of standard hemicolectomy rather than being oncologically necessary [10]. Ueno et al. also reported that lymph node metastasis beyond 10 cm from the tumor occurred in only 0.1% of cases [33]. In the present study, no lymph node recurrence was observed in the ICA or RCA in the TC group. But lymph node metastasis in the RCA territories was observed in two cases (#211 1.8%, #212 1.8%). There were reports suggesting that PETCT was associated with improved prognostic outcomes in surgical treatment [34] and that indocyanine green (ICG) can increase lymph node retrieval rates [35]. The preoperative use of PETCT to assess lymph node metastasis in the RCA, as well as the intraoperative use of ICG, may help guide the choice between exRHC and TC. Consistent with previous reports, lymphadenectomy limited to the MCA region was considered sufficient for mid-transverse colon cancer.

The strength of our study was that the analysis was limited to stage II/III patients who underwent D3 lymph node dissection with central ligation of the MCA. This allowed for a standardized comparison of surgical techniques across the cohort. Two reports have described the extent of lymphadenectomy during the surgical procedure for mid-transverse colon cancer. Iguchi et al. reported that patients in the TC had significantly shorter operative times, lower rates of CD grade ≥ 3 complications, and significantly lower rates of anastomotic leakage than those in the exRHC group. However, regarding background characteristics, more than half of the TC group were T1/2 (TC, 58.8% vs. exRHC, 31.4%) and stage I (50% vs. 31.4%), resulting in more than half undergoing D1/2 lymph node dissection (50% vs. 17.1%, *p* = 0.039). Matsuda et al. reported higher postoperative complication rates in the TC group than in the exRHC group, although not statistically significant; the rate of D3 lymph node dissection was lower in the TC group than in the exRHC group (73.5% vs. 91.6%, *p* = 0.226). Both reports stated that there were no significant differences in the long-term outcomes, such as the RFS and OS. However, since both reports showed differences in background factors, it was difficult to directly compare short- and long-term outcomes. Therefore, in the present study, we limited our analysis to cases with D3 lymph node dissection only.

In the present study, 3-year RFS and OS were comparable between the exRHC and TC groups. These findings are consistent with previous reports in the literature. Elkomos et al. demonstrated that although exRHC was associated with prolonged operative time and a higher number of harvested lymph nodes, its oncological outcomes were comparable to those achieved with TC [31]. Similarly, Chon et al., in a large cohort study, demonstrated equivalent RFS and OS between patients treated with TC and exRHC, suggesting no survival disadvantage with the more limited resection [4]. In addition, Van Rongen and Leijssen et al. compared TC and exRHC and suggested that TC may be a more appropriate suitable surgical option in selected patients, given its comparable oncological efficacy and potentially reduced invasiveness [7, 36]. Furthermore, several studies have reported that TC provides long-term oncological outcomes similar to those of RHC, without affecting recurrence patterns or the distribution of recurrent disease [4, 10, 37]. Taken together, these findings support the notion that TC can be considered an oncologically sound alternative to exRHC for mid-transverse colon cancer.

Several limitations of the present study should be carefully considered. First, this study was a retrospective analysis, and advanced tumors of mid-transverse colon cancer are relatively uncommon, which inherently limits the number of eligible patients. Moreover, by including only cases that received D3 lymph node dissection, the study cohort was limited in size. This limitation may have affected the strength of the statistical analyses and the applicability of the results to a broader patient population. Second, the choice of the surgical procedure was left to the discretion of the operating surgeon. This introduces the possibility of selection bias, as the choice between TC and exRHC may have been influenced by the individual surgeon’s experience, preference, or perceived complexity of each case. The distribution of cases between the groups was skewed, with a higher proportion of c-Stage III patients in the exRHC group, and no cases of postoperative ileus were observed in the TC group. In addition, preoperative clinical factors such as the colonoscopic passage rate and the incidence of obstructive colitis were not available. Although no statistically significant differences were observed, patients diagnosed with c-Stage III disease—that is, those with more advanced disease—may have been more likely to undergo exRHC. The potential impact of this selection bias, including an increased risk of postoperative complications and poorer oncological outcomes, should be taken into consideration. Therefore, the results should be interpreted with caution, and further studies with balanced baseline characteristics are warranted. Third, there was variability in the TC technique itself. Specifically, whether mobilization included both the hepatic and splenic flexures or only one of these structures differed among patients, resulting in a lack of complete uniformity in the procedure. Fourth, the definition and handling of the RCA lymph nodes in the TC group were not clearly established. It remains unclear whether the RCA lymph nodes was truly excluded from the lymphadenectomy in the TC group. Given that anatomical variations are common, the RCA may arise directly from the superior mesenteric artery or branch from the MCA. In cases where the RCA branches from the MCA, the extent of lymphadenectomy performed during TC could potentially include part of the RCA lymph nodes. Fifth, short-term postoperative readmission rates were not available across all participating institutions. Sixth, in terms of short-term complications, only one case of diarrhea was observed in the TC group. TC preserves the terminal ileum, ascending colon, and ileocecal valve, potentially reducing injury to the autonomic nerve plexus. In contrast, exRHC often involves plexus injury or resection, which may contribute to postoperative diarrhea and bowel dysfunction [28, 29]. Consistent with these anatomical and functional considerations, postoperative functional impairment, such as diarrhea or other bowel dysfunction, may have influenced the ability to initiate or complete adjuvant chemotherapy. These factors may have influenced surgical outcomes and limited the ability to draw definitive conclusions. To address these limitations, a well-designed, large-scale, randomized clinical trial is currently underway by the Japan Clinical Oncology Group (JCOG2304 trial), and the results of this trial are highly anticipated, as they are expected to provide more robust evidence. 

## Conclusions

In D3 lymph node dissection with central ligation of the MCA for advanced mid-transverse colon cancer, exRHC was associated with a significantly increased incidence of postoperative ileus and a prolonged hospital stay. These findings suggest that TC may be a favorable surgical option for mid-transverse colon cancer.

## Supplementary Information

Below is the link to the electronic supplementary material.

ESM 1 Supplementary Material 1 (DOCX 39.1 KB)

## Data Availability

The datasets generated and/or analyzed during the current study are not publicly available due to the sensitive nature of patient information. Access to the data can be requested from the corresponding author and will be provided under reasonable conditions, subject to approval by the institutional review board and compliance with applicable privacy regulations. All shared data will be fully anonymized to protect patient confidentiality.

## References

[1] Schlachta CM, Mamazza J, Poulin EC (2007) Are transverse colon cancers suitable for laparoscopic resection? Surg Endosc 21(3):396–399 10.1007/s00464-006-9042-6 17103274

[2] de’Angelis N, Alghamdi S, Renda A, Azoulay D, Brunetti F (2015) Initial experience of robotic versus laparoscopic colectomy for transverse colon cancer: a matched case-control study. World J Surg Oncol. 10.1186/s12957-015-0708-1 PMC4598969 26452727

[3] Wray CM, Ziogas A, Hinojosa MW, Le H, Stamos MJ, Zell JA (2009) Tumor subsite location within the colon is prognostic for survival after colon cancer diagnosis. Dis Colon Rectum 52(8):1359–1366 10.1007/DCR.0b013e3181a7b7de 19617745

[4] Chong CS, Huh JW, Oh BY, Park YA, Cho YB, Yun SH et al (2016) Operative method for transverse colon carcinoma: transverse colectomy versus extended colectomy. Dis Colon Rectum 59(7):630–639 10.1097/DCR.0000000000000619 27270515

[5] Milone M, Degiuli M, Allaix ME, Ammirati CA, Anania G, Barberis A et al (2020) Mid-transverse colon cancer and extended versus transverse colectomy: results of the Italian society of surgical oncology colorectal cancer network (SICO CCN) multicenter collaborative study. Eur J Surg Oncol 46(9):1683–1688 10.1016/j.ejso.2020.01.006 32220542

[6] Sun R, Zhang G, Sun X, Niu B, Zhou J, Cong L et al (2023) Comparing the techniques and outcomes of laparoscopic transverse colectomy to laparoscopic hemicolectomy in mid-transverse colon cancer resection. Front Surg. 10.3389/fsurg.2022.1012947 PMC9852304 36684238

[7] Leijssen LGJ, Dinaux AM, Amri R, Kunitake H, Bordeianou LG, Berger DL (2018) A transverse colectomy is as safe as an extended right or left colectomy for mid-transverse colon cancer. World J Surg 42(10):3381–3389 10.1007/s00268-018-4582-1 29556881

[8] Park H, Lee J, Lee SY, Kim CH, Kim HR (2021) Distribution of lymph node metastasis and oncological outcomes of mid-transverse colon cancer: extended versus transverse colectomy. Colorectal Dis 23(8):2007–2013 10.1111/codi.15659 33793058

[9] Almoregy AS, Osman G, Sharafedeen M, Alattar AZ (2021) Laparoscopic extended hemicolectomy vs laparoscopic transverse colectomy for management of mid-transverse colon cancer—which is the optimal surgical approach? World 14(3):216

[10] Fukuoka H, Fukunaga Y, Nagasaki T, Akiyoshi T, Konishi T, Nagayama S et al (2022) Lymph node mapping in transverse colon cancer treated using laparoscopic colectomy with D3 lymph node dissection. Dis Colon Rectum 65(3):340–352 10.1097/DCR.0000000000002108 35138285

[11] Hashiguchi Y, Muro K, Saito Y, Ito Y, Ajioka Y, Hamaguchi T et al (2020) Japanese Society for Cancer of the Colon and Rectum (JSCCR) guidelines 2019 for the treatment of colorectal cancer. Int J Clin Oncol 25(1):1–42 10.1007/s10147-019-01485-z PMC6946738 31203527

[12] Balciscueta Z, Balciscueta I, Uribe N, Pellino G, Frasson M, García-Granero E et al (2021) D3-lymphadenectomy enhances oncological clearance in patients with right colon cancer. Results of a meta-analysis. Eur J Surg Oncol 47(7):1541–1551 10.1016/j.ejso.2021.02.020 33676793

[13] Kim HJ, Lee IK, Lee YS, Kang WK, Park JK, Oh ST et al (2009) A comparative study on the short-term clinicopathologic outcomes of laparoscopic surgery versus conventional open surgery for transverse colon cancer. Surg Endosc 23(8):1812–1817 10.1007/s00464-009-0348-z 19263150

[14] Fléjou JF (2011) [WHO Classification of digestive tumors: the fourth edition]. Ann Pathol 31(5 Suppl):S27–31. https://pubmed.ncbi.nlm.nih.gov/22054452/. Accessed 7 July 2026 10.1016/j.annpat.2011.08.001 22054452

[15] Brierley J, Gospodarowicz MK, Wittekind C (2017) TNM classification of malignant tumors international union against cancer, 8th edn. Oxford, England: Wiley Oesphagus including oesophagogas

[16] Dindo D, Demartines N, Clavien PA (2004) Classification of surgical complications: a new proposal with evaluation in a cohort of 6336 patients and results of a survey. Ann Surg 240(2):205–213 10.1097/01.sla.0000133083.54934.ae PMC1360123 15273542

[17] Yin J, Salem ME, Dixon JG, Jin Z, Cohen R, Degramont A et al (2022) Reevaluating disease-free survival as an endpoint vs overall survival in stage III adjuvant colon cancer trials. J Natl Cancer Inst 114(1):60–67 10.1093/jnci/djab187 PMC8755492 34505880

[18] Amri R, Klos CL, Bordeianou L, Berger DL (2016) The prognostic value of lymph node ratio in colon cancer is independent of resection length. Am J Surg 212(2):251–257 10.1016/j.amjsurg.2015.10.037 27156798

[19] Stracci F, Bianconi F, Leite S, Liso A, La Rosa F, Lancellotta V et al (2016) Linking surgical specimen length and examined lymph nodes in colorectal cancer patients. Eur J Surg Oncol 42(2):260–265 10.1016/j.ejso.2015.11.017 26723169

[20] Toyota S, Ohta H, Anazawa S (1995) Rationale for extent of lymph node dissection for right colon cancer. Dis Colon Rectum 38(7):705–711 10.1007/BF02048026 7607029

[21] Cooper CR, Feingold DL (2019) Laparoscopic colectomy for colonic malignancy: review of literature. Ann Laparosc Endosc Surg 4:3

[22] Søndenaa K, Quirke P, Hohenberger W, Sugihara K, Kobayashi H, Kessler H et al (2014) The rationale behind complete mesocolic excision (CME) and a central vascular ligation for colon cancer in open and laparoscopic surgery: proceedings of a consensus conference. Int J Colorectal Dis 29(4):419–428 10.1007/s00384-013-1818-2 24477788

[23] Chow TC, Wong CK, Si AL, Ng AS, Kong CK (2021) Retrospective analysis of a single institute’s experience of transverse colectomy vs extended colectomy for the management of mid-transverse colon cancer. Surg Pract 25(2):69–77

[24] Iguchi K, Numata M, Shiozawa M, Kazama K, Sawazaki S, Katayama Y et al (2022) Laparoscopic extended right hemicolectomy versus laparoscopic transverse colectomy for mid-transverse colon cancer: a multicenter retrospective study from Kanagawa Yokohama Colorectal Cancer (KYCC) study group. Int J Colorectal Dis 37(5):1011–1019 10.1007/s00384-022-04128-4 35384494

[25] Matsuda T, Sumi Y, Yamashita K, Hasegawa H, Yamamoto M, Matsuda Y et al (2018) Optimal surgery for mid-transverse colon cancer: laparoscopic extended right hemicolectomy versus laparoscopic transverse colectomy. World J Surg 42(10):3398–3404 10.1007/s00268-018-4612-z 29610931

[26] Ibañez N, Abrisqueta J, Luján J, Hernández Q, Parrilla P (2017) Isoperistaltic versus antiperistaltic side-to-side anastomosis after right laparoscopic hemicolectomy for cancer (ISOVANTI) trial: study protocol for a randomised clinical trial. Int J Colorectal Dis 32(9):1349–1356 10.1007/s00384-017-2840-6 28634703

[27] Parnasa SY, Shweiki A, Pinhasov D, Abu Salem S, Gefen R, Mizrahi I et al (2025) Right extended hemicolectomy with ileo-descending anastomosis is associated with prolonged postoperative ileus and longer hospital stay compared to right or left hemicolectomy. Int J Colorectal Dis 40(1):134 10.1007/s00384-025-04926-6 PMC12134037 40461834

[28] Bertelsen CA, Larsen HM, Neuenschwander AU, Laurberg S, Kristensen B, Emmertsen KJ (2018) Long-term functional outcome after right-sided complete mesocolic excision compared with conventional colon cancer surgery: a population-based questionnaire study. Dis Colon Rectum 61(9):1063–1072 10.1097/DCR.0000000000001154 30086055

[29] Lee KM, Baek SJ, Kwak JM, Kim J, Kim SH (2020) Bowel function and quality of life after minimally invasive colectomy with D3 lymphadenectomy for right-sided colon adenocarcinoma. World J Gastroenterol 26(33):4972–4982 10.3748/wjg.v26.i33.4972 PMC7476173 32952343

[30] Thorsen Y, Stimec B, Andersen SN, Lindstrom JC, Pfeffer F, Oresland T et al (2016) Bowel function and quality of life after superior mesenteric nerve plexus transection in right colectomy with D3 extended mesenterectomy. Tech Coloproctol 20(7):445–453 10.1007/s10151-016-1466-y 27137207

[31] Elkomos BE, Alkomos PE, Alkomos MF, Ahmed S, Baqar SO, Bhatti MFR et al (2024) Optimal surgical approach for mid-transverse colon cancer: a systematic review and meta-analysis. Int J Clin Oncol 29(6):706–715 10.1007/s10147-024-02486-3 PMC11130047 38679628

[32] Chang GJ, Rodriguez-Bigas MA, Skibber JM, Moyer VA (2007) Lymph node evaluation and survival after curative resection of colon cancer: systematic review. J Natl Cancer Inst 99(6):433–441 10.1093/jnci/djk092 17374833

[33] Ueno H, Hase K, Shiomi A, Shiozawa M, Ito M, Sato T et al (2023) Optimal bowel resection margin in colon cancer surgery: prospective multicentre cohort study with lymph node and feeding artery mapping. Lancet Reg Health West Pac 33. https://pubmed.ncbi.nlm.nih.gov/37181532/. Accessed 7 July 2026 10.1016/j.lanwpc.2022.100680 PMC10166781 37181532

[34] Liang JT, Chen TC, Liao YT, Huang J, Hung JS (2024) Impact of positron-emission tomography on the surgical treatment of locoregionally recurrent colorectal cancer. Asian J Surg 47(2):923–932 10.1016/j.asjsur.2023.10.109 38042659

[35] Sun Y, Tang Z, Deng Y, Xu Z, Chen Z, Huang S et al (2024) Safety and efficacy of indocyanine green fluorescence imaging-guided laparoscopic para-aortic lymphadenectomy for left-sided colorectal cancer: preliminary results of a case-matched study. Asian J Surg 47(11):4744–4751 10.1016/j.asjsur.2024.04.027 38664189

[36] Van Rongen I, Damhuis RA, Van Der Hoeven JA, Plaisier PW (2013) Comparison of extended hemicolectomy versus transverse colectomy in patients with cancer of the transverse colon. Acta Chir Belg 113(2):107–11 23741929

[37] Yamamoto M, Okuda J, Tanaka K, Kondo K, Tanigawa N, Uchiyama K (2012) Clinical outcomes of laparoscopic surgery for advanced transverse and descending colon cancer: a single-center experience. Surg Endosc 26(6):1566–1572 10.1007/s00464-011-2071-9 PMC3351590 22179459

