# Supplementary material for: A comparison of extended right hemicolectomy and transverse colectomy with D3 lymph node dissection for pathological stage II/III advanced mid-transverse colon cancer
Source: Int J Colorectal Dis. 2026 Jul 29;41(1):171. doi: 10.1007/s00384-026-05202-x (PMC13710656; doi:10.1007/s00384-026-05202-x)
Supplement: Supplementary file 1 [file 384_2026_5202_MOESM1_ESM.docx]

**SUPPLEMENTARY TABLE 1. Univariate** **and multivariate analyses of risk factors for the postoperative ileus (n = 83)**

| *Variable* | *Univariate analysis* | | |  | *Multivariate analysis* | |
| --- | --- | --- | --- | --- | --- | --- |
|  | *Postoperative ileus, n = 83* | | | *p* | *OR (95% CI)* | *p* |
| Age, years |  |  | | 1 | 0.352 (0.057–2.170) | 0.261 |
| <70 | 3/ 27 (11.1%) | | |  |  |  |
| ≥70 | 6/ 56 (10.7%) | | |  |  |  |
| BMI, kg/m^2^ |  |  | | 0.342 | 5.87e-09 (0–Inf) | 0.996 |
| <25.0 | 9/ 70 (12.9%) | | |  |  |  |
| ≥25.0 | 0/ 13 (0%) | | |  |  |  |
| ASA-PS score |  | |  | 0.567 | 2.350 (0.162–34.10) | 0.531 |
| I/ II | 8/ 76 (10.5%) | | |  |  |  |
| III/ IV | 1/ 7 (14.3%) | | |  |  |  |
| c-Stage |  | | | 0.728 | 0.335 (0.062–1.820) | 0.205 |
| I/ II | 4/ 32 (12.5%) | | |  |  |  |
| IIII/ IV | 5/ 51 (9.8%) | | |  |  |  |
| Surgical approaches |  | |  | 1 | 0.931 (0.078–11.10) | 0.955 |
| Open surgery | 1/ 9 (11.1%) | | |  |  |  |
| Conventional laparoscopic surgery, Robot-assisted laparoscopic surgery | 8/ 74 (10.8%) | | |  |  |  |
| Surgical procedures |  | |  | 0.028 | 1.95e+08 (0–Inf) | 0.995 |
| exRHC | 9/ 56 (16.1%) | | |  |  |  |
| TC | 0/ 27 (0%) | | |  |  |  |

BMI, body mass index; ASA, American Society of Anesthesiologists Physical Status Classification System; exRHC, extended right hemicolectomy; TC, transverse colectomy; OR, odds ratio; CI, confidence interval

**SUPPLEMENTARY TABLE 2. Univariate analysis of risk factors for the postoperative ileus after PSM (n = 54)**

| *Variable* | *Univariate analysis* | | |  |
| --- | --- | --- | --- | --- |
|  | *Postoperative ileus, n = 54* | | | *p* |
| Age, years |  |  | | 0.652 |
| <70 | 1/ 17 (5.9%) | | |  |
| ≥70 | 5/ 37 (13.5%) | | |  |
| BMI, kg/m^2^ |  |  | | 0.574 |
| <25.0 | 6/ 45 (13.3%) | | |  |
| ≥25.0 | 0/ 9 (0%) | | |  |
| ASA-PS score |  | |  | 0.385 |
| I/ II | 5/ 50 (10%) | | |  |
| III/ IV | 1/ 4 (25%) | | |  |
| c-Stage |  | | | 1 |
| I/ II | 4/ 32 (12.5%) | | |  |
| IIII/ IV | 2/ 22 (9.1%) | | |  |
| Surgical approaches |  | |  | 1 |
| Open surgery | 0/ 4 (0%) | | |  |
| Conventional laparoscopic surgery, Robot-assisted laparoscopic surgery | 6/ 50 (12.0%) | | |  |
| Surgical procedures |  | |  | 0.023 |
| exRHC | 6/ 27 (22.2%) | | |  |
| TC | 0/ 27 (0%) | | |  |

BMI, body mass index; ASA, American Society of Anesthesiologists Physical Status Classification System;

exRHC, extended right hemicolectomy; TC, transverse colectomy; OR, odds ratio; CI, confidence interval
